# Financial Implications of the Updated ACGME Requirements for Emergency Medicine Residency Programs

**DOI:** 10.1002/aet2.70245

**Published:** 2026-09-06

**Authors:** Douglas McGee, Mary Jo Wagner

**Affiliations:** ^1^ Sidney Kimmel Medical College, Thomas Jefferson University Philadelphia Pennsylvania USA; ^2^ CMU Medical Education Partners, Covenant HealthCare College of Medicine at Central Michigan University (CMU) Saginaw Michigan USA

## Abstract

The newly posted 2026 Emergency Medicine (EM) Program Requirements by the Accreditation Council of Graduate Medical Education (ACGME) creates both financial challenges and opportunities for programs and hospitals. The complexity of the federal government funding of GME through Centers for Medicare and Medicaid Services (CMS) makes it difficult for program and institutional Graduate Medical Education (GME) leaders to identify the financial implications of changes in clinical EM operations and curriculum design. Institutional initiation of a change in program format is best implemented with an understanding of the hospital's financial priorities. In this concept paper, we discuss steps to clearly identify the financial impact of the EM residency program format on CMS funding and program complement size. We also provide clarifications on the new ACGME required programmatic changes and their impact on EM residency funding.

## 1 Introduction

The recent changes posted for the major revision of the Accreditation Council for Graduate Medical Education (ACGME) program requirements for Emergency Medicine (EM) have financial implications that may impact program planning and implementation. We describe financial challenges and opportunities that the program requirements might present as EM residency programs work toward changes over the next few years. The funding concepts discussed will require local adaptation and execution to prioritize the educational goals of the suggested changes to the EM residency curriculum. Potential increases in governmental reimbursement if the 4‐year training format was mandated by the ACGME and changes to program complements have the biggest impact on program financial performance. As the ACGME did not mandate the 4‐year structure, some of the described funding and financial implications will only apply if a program contemplates a change in format. But with the ACGME indication of a pilot program to determine whether the training length should change in the future, we felt it important to explain this to the community. Other new requirements could have financial implications depending on local implementation. This paper does not consider the academic consequences of suggested strategies but rather describes the potential fiscal impact of program changes related to the requirements. What is suggested here can be used as an outline for advancing the educational opportunities in the new requirements.

## 2 Understanding the Fundamentals of GME Financing

A review of some fundamental Centers for Medicare and Medicaid Services (CMS) policies and practices for GME financing will help frame this discussion. CMS Funding for residents/fellows flows to each teaching hospital through the Medicare payment system and is composed of two parts: Direct Graduate Medical Education (DGME) [1] and Indirect Medical Education (IME) [2]. DGME is intended to support Medicare's portion of the expense of resident/fellows' salary and benefits, teaching cost of faculty, and GME infrastructure such as the library and simulation center (Figure 1). IME is designed to offset indirect patient costs related to GME requirements and teaching hospital operating costs including the increase in complexity of patients, standby services cost (burn center, trauma teams), and learner inefficiencies. This indirect cost is added to the diagnostic‐related group (DRG) reimbursement in proportion to the Medicare patients cared for at a hospital (Figure 2). Generally, the DGME is one‐third and the IME is two‐thirds of the total CMS GME funding, though this can vary among hospitals for a variety of reasons beyond the scope of this document (Figure 3). Further background for the details of federal government funding in GME can be found in other sources [3, 4].

**FIGURE 1:**
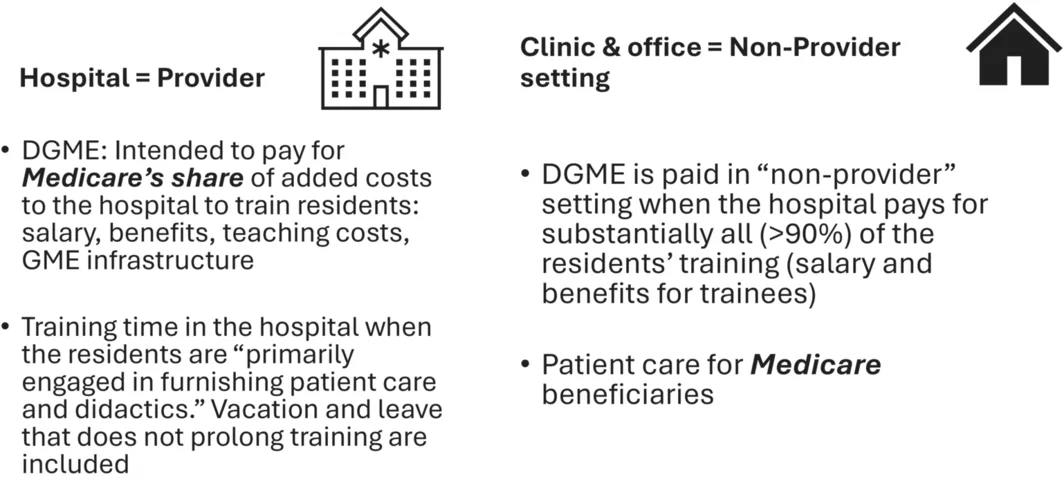
Direct Graduate Medical Education (DGME) CMS funding.

**FIGURE 2:**
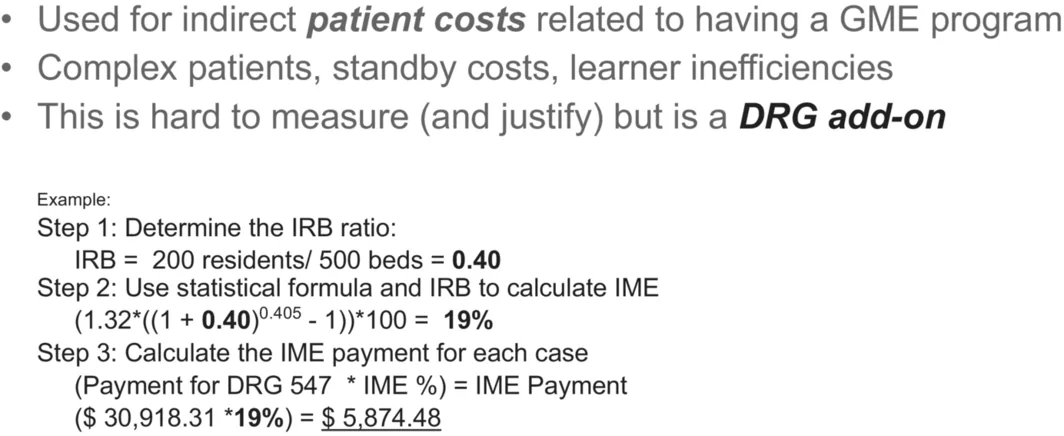
Indirect Medical Education (IME) CMS funding.

**FIGURE 3:**
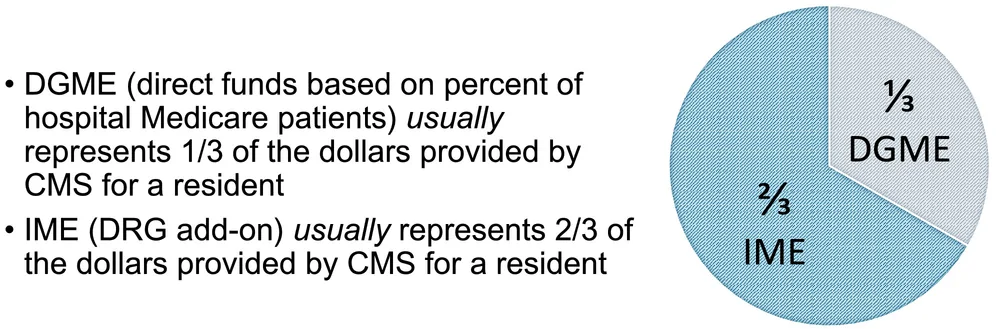
Total CMS dollars.

Another determination of the total financial support for GME trainees was established by CMS in 1997 when the total number of residents training at a hospital became the total reimbursed, thus the “cap” of allowed trainee funding [3]. There have been occasional opportunities to increase this capped CMS financing, but most hospitals are training more residents and fellows than CMS reimbursement support provides. The number of residents and fellows is not capped; the reimbursement is capped.

Hospitals are reimbursed by CMS some or all the DGME costs based on the Initial Residency Period (IRP) for each individual trainee. The assigned IRP length equals the training years needed to seek *primary* board certification in a given specialty [5]. The minimum number of years determines the IRP duration for which CMS pays 100% of the DGME and 100% IME. When a medical student graduates and matriculates into residency training, the very first cost report of the hospital in which they train sets their IRP in most cases (there are some exceptions). Moreover, once an IRP is set for an individual trainee, it is never changed. If a resident/fellow extends training by starting in a Transitional year, transferring to a different specialty, or extends GME training to do a fellowship, then only 50% of the DGME is paid for those additional years of training. The complete IME funding per resident, associated directly with patient care, remains intact regardless of additional training.

The duration of time for EM's IRP is set by CMS at 3 years even though the American Board of Emergency Medicine (ABEM) now requires residents to complete the program they started, regardless of duration, before being eligible for the ABEM examination process leading to board certification [6]. ABEM requires residents in 3‐year programs to complete the 3 years and residents in 4‐year programs to complete all the 4 years. Currently, the fourth‐year EM residents are training past the 3‐year IRP, and the “Direct GME (DGME)” portion of CMS reimbursement is reduced by 50%. Fourth‐year EM residents are eligible for the full 100% of the “Indirect Medical Education (IME)” portion of CMS funding. When considering the current CMS rules and ACGME EM requirements, an institution at or under their resident cap is reimbursed annually over time 100% DGME and IME for each typical EM resident in a 3‐year program or 96% for each resident over the training time in a 4‐year program (Figure 4). When considered as part of an EM program, the difference in cost is decreased to approximately 2% of the annual program cost (Figure 5).

**FIGURE 4:**
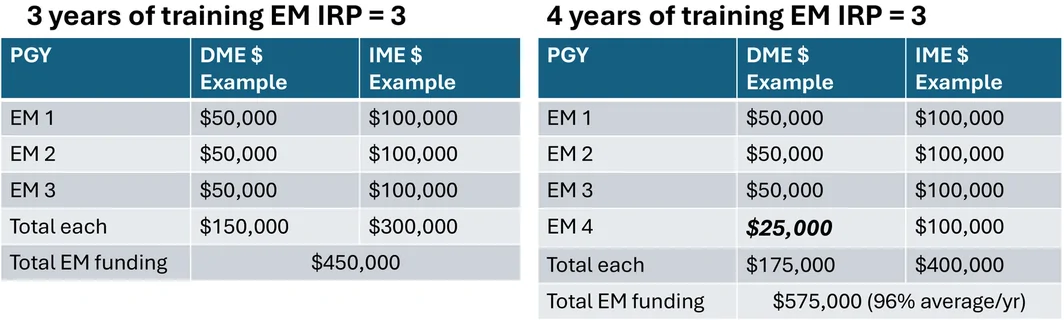
Three‐year IRP, 4‐year program—One resident. CMS reimbursement rates vary widely between hospitals. This presumes the typical ratio of reimbursement which is one‐third DGME and two‐thirds IME and uses rounded dollars to make calculations clear.

**FIGURE 5:**
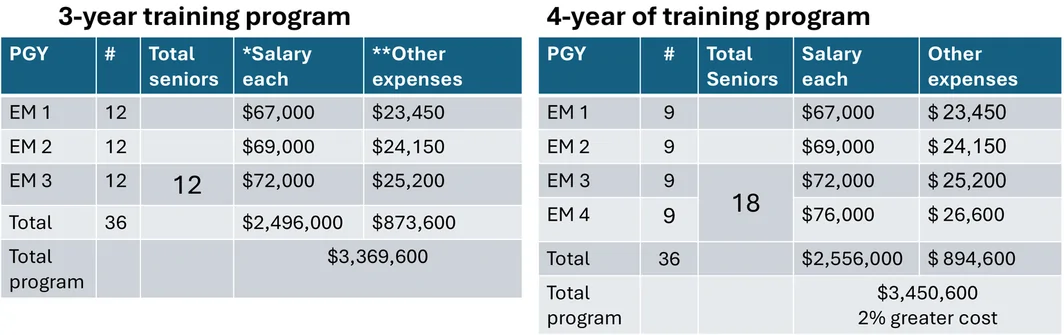
Three‐year program to 4‐year program—Program cost. *AAMC mean 2024. **Benefits/liability/other—35%.

CMS is required by statute to set an IRP to equal the years required for “initial board eligibility” [3]. GME leaders (including these authors) from the American College of Emergency Physicians (ACEP) met with representatives of CMS and reaffirmed that if ACGME‐accredited EM programs are required to be 4 years in duration, CMS will follow standard policy and adjust the IRP for EM to 4 years. If ACGME were to require a 4‐year training minimum, all residents, at all training levels, in all programs, will be eligible for the full 4 years of funding of DGME and IME from CMS. Current 4‐year programs will then be eligible for full CMS funding, and current 3‐year programs transitioning to a 4‐year format will be eligible for full CMS funding for the fourth year. If the ACGME does not require the change to a 4‐year format, the IRP for EM will remain at 3 years. As demonstrated in Figure 6, funding for a 4‐year program will remain at a high rate, dependent upon an institution's DGME and IME reimbursement, so the same considerations below should be considered to determine the financial cost or benefit for transitioning to a 4‐year program.

**FIGURE 6:**
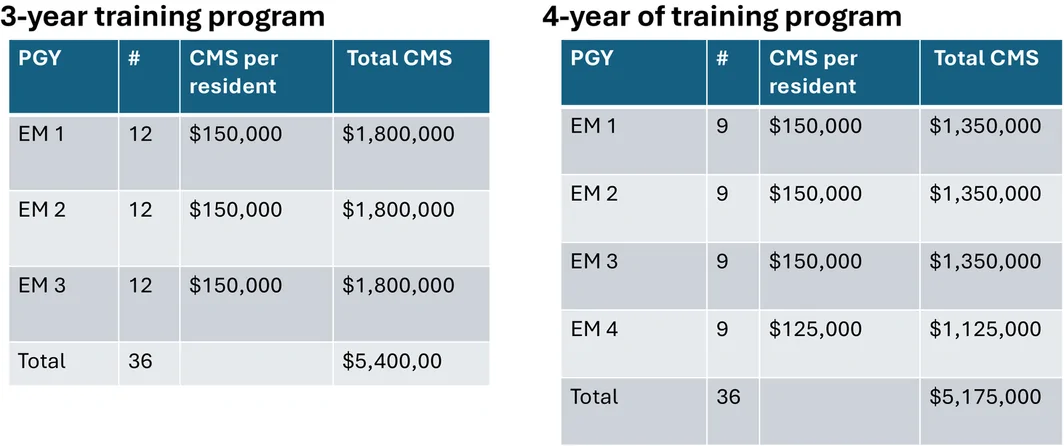
Three‐year program to 4‐year program—Program reimbursement. AAMC mean 2024. Benefits/liability/other—35%.

To date, there is no outline for the pilot program, but some residencies may select to change from one format to another if given the opportunity. To understand the financial implications of changing from one training length to another, adding new ED‐based experiences or providing outside EM experiences, consider the following steps (Figure 7).

**FIGURE 7:**
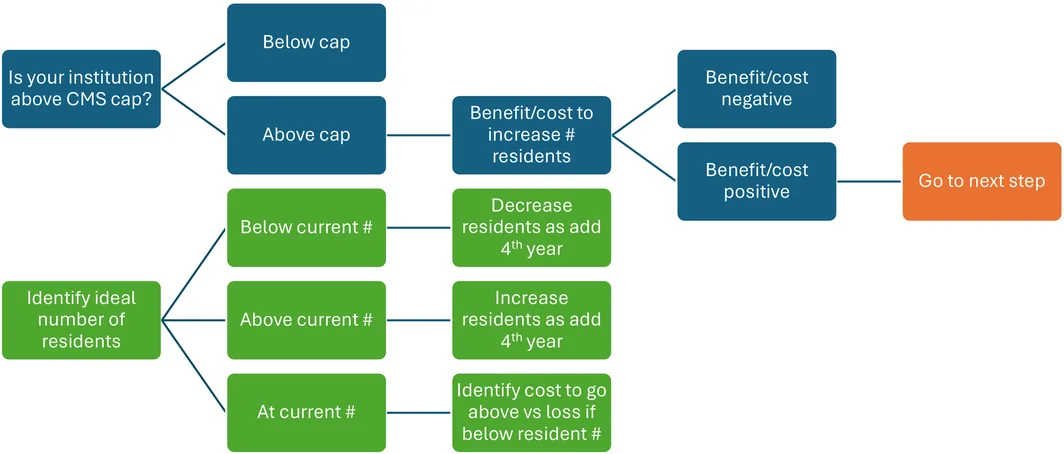
Flow chart for changing from 3‐year program to 4‐year program.

### 2.1 Step 1: Determine if Your Institution Is Below, at, or Above the Cap, for CMS Reimbursement

#### 2.1.1 Institutions Building or Under a Cap

Institutions building a CMS cap, or under the CMS cap, will receive additional CMS revenue if the 4‐year format becomes required in the future.

1. Institutions now sponsoring a 4‐year program will see DGME funding for the final year increase from 50% to 100%. IME funding will continue at 100%.
2. Institutions transitioning from a 3‐year program to a 4‐year program will be fully reimbursed for 100% of both DGME and IME for residents in the fourth year.
3. Legacy 4 programs and new 4‐year programs will receive full funding for program expansions to the limits of the institutional cap.

#### 2.1.2 Institutions Over the CMS Cap

Institutions over the CMS cap are not now fully reimbursed for GME training regardless of the program type or discipline. However, the potential future change in IRP will create potential opportunities.

1. Institutions managing their GME portfolio and attempting to reduce or prioritize programs would be able to consider 4‐year EM programs as “fully reimbursed” rather than a program past the IRP.
2. Programs rotating EM residents to participating sites (PS) that have CMS reimbursement available can negotiate full reimbursement from the PS, who will be fully reimbursed by CMS for EM residents rotating to the PS.

### 2.2 Step 2: Consider the Impact on Salary, Benefits, and Other Scalar Program Expenses for Complement Changes

Financial implications of program expansion or contraction are evaluated locally in a manner that considers incremental expense or revenue changes. Residency leaders will need to work with department and institutional financial colleagues to evaluate considered changes if the pilot leads to a requirement for a 4‐year training length. Three‐year programs transitioning to the 4‐year format have three options available that could impact program expenses. The ACGME does not require that each program class be the same size. For the convenience of scheduling, a program transitioning to a 4‐year format may choose to have a total complement divisible by four rather than three. Each program may choose to save the cost of some resident salaries with fewer residents during the transition, or have more residents and their cost, but avoid gaps in service. The authors anticipate the ACGME will provide some flexibility for resident complement changes during this transition if required. We have chosen to provide examples using a total complement starting with 36 residents to demonstrate the changes a program may make if the length of training is required to be 4 years.

#### 2.2.1 Neutral Complement

Should the program desire the same number of total residents in the program, the current total complement can be divided by four rather than three. In this scenario, there will be fewer residents in each of the original three classes over time with less salary costs though this will be offset by a typically higher salary cost for fourth year residents. If the program chooses to remain “complement neutral,” the salary expense impact will be minimal. CMS revenue will remain stable unless the program adopts other curricular changes that impact CMS claimable time. All other scalar program costs such as GME leader and Core Faculty support, and coordinator expenses will be the same if the complement is unchanged. (See Table 1 for an example of the neutral complement transition.)

**TABLE 1:** Neutral complement transition example.

Academic year	ACGME‐approved complement	PGY1	PGY2	PGY3	PGY1	PGY2	PGY3	PGY4	Total residents in program
2027–2028	36	12	12	12					36
2028–2029	36		12	12	12				36
2029–2030	36			12	12	12			36
2030–2031	36				9	12	12		33
2031–2032^a^	36				9	9	12	12	42
2032–2033^a^	36				9	9	9	12	39
2033–2034	36				9	9	9	9	36

*Note:* Pink—All residents with a 3‐year IRP. Yellow—Some residents with a 3‐year IRP and some with a 4‐year IRP. Blue—All residents with a 4‐year IRP.

^a^ Programs will need to request complement increase from the ACGME for transitional years.

#### 2.2.2 Reduce Complement

A decision to create a smaller sized program would save the hospital salary and expenses for residents. Should a reduced complement require operational and staffing changes, the hospital will need to consider any additional “back fill” expenses for attending physicians, physician assistants, or nurse practitioners. Some EM requirements are scalar (program leadership and coordinator support and the number of required core faculty), and program complement reductions below the requirement threshold ranges in these areas may allow for additional expense reductions. (See Table 2 for an example of the reduced complement transition.)

**TABLE 2:** Reduced complement transition example.

Academic year	ACGME‐approved complement	PGY1	PGY2	PGY3	PGY1	PGY2	PGY3	PGY4	Total residents in program
2027–2028	36	12	12	12					36
2028–2029	36		12	12	10				34
2029–2030	36			12	9	10			31
2030–2031	36				9	9	10		28
2031–2032	36				8	9	9	10	32
2032–2033	36				8	8	9	9	34
2033–2034	36				8	8	8	9	33
2034–2035	36				8	8	8	8	32

*Note:* Pink—All residents with a 3‐year IRP. Yellow—Some residents with a 3‐year IRP and some with a 4‐year IRP. Blue—All residents with a 4‐year IRP.

#### 2.2.3 Increase Complement

The decision to grow the program will be made locally using the financial analysis engaged for any proposed program complement increase. Because program leader, core faculty, and coordinator expenses are scalar within complement ranges, a slight increase in complement may not add any expense to the program in these areas. As fourth year residents will be eligible for full reimbursement by CMS, this class may result in increased CMS revenue for the program, fully offsetting any potential salary or other program increases. (See Table 3 for an example of the increased complement transition).

Consider the impact on overall staffing in the Emergency Department (ED) when evaluating a program complement increase (Figure 8). Additional residents might require a redistribution of faculty, residents, and Advanced Practice Clinicians (APC: physician assistants and nurse practitioners) staffing. Generally, APC salaries are higher than resident salaries; residents tend to train more hours than APCs are scheduled to work, and the resident component of the workforce is more stable with less unfilled positions or turnover. Replacing APC expenses with residents that are reimbursable by CMS might result in substantial cost savings [7, 8].

**TABLE 3:** Increased complement transition example.

Academic year	ACGME‐approved complement	PGY1	PGY2	PGY3	PGY1	PGY2	PGY3	PGY4	Total residents in program
2027–2028	36	12	12	12					36
2028–2029	36		12	12	12				36
2029–2030	36			12	12	12			36
2030–2031	36				12	12	12		36
2031–2032^a^	36				12	12	12	12	48

*Note:* Pink—All residents with a 3‐year IRP. Yellow—Some residents with a 3‐year IRP and some with a 4‐year IRP. Blue—All residents with a 4‐year IRP.

^a^ Programs will need to request compliment increase from the ACGME.

**FIGURE 8:**
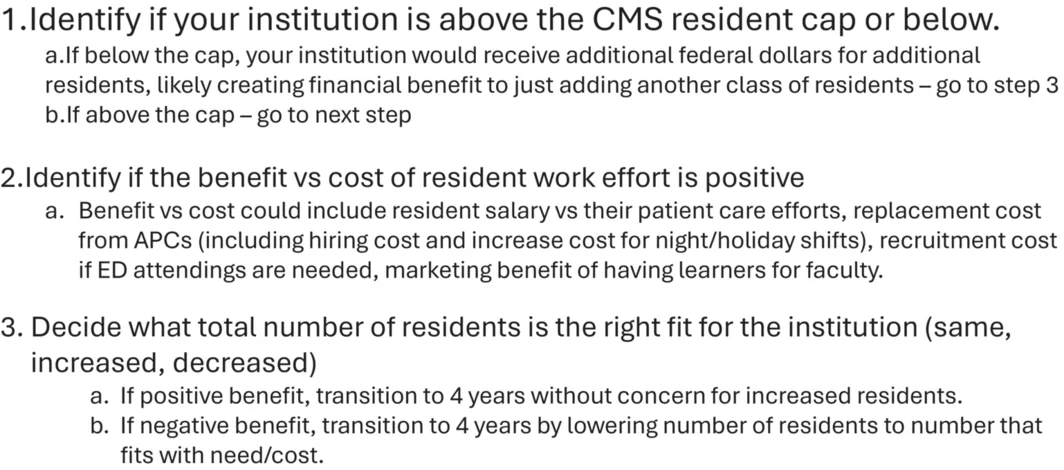
Steps for changing from 3‐year program to 4‐year program.

## 3 Lever the Senior Resident Contribution While Maximizing Educational Opportunities

Additional hours staffed by a more experienced resident further enhance the potential billable case volume in the ED. A senior resident supervising junior residents might enhance the ability of the attending staff to be available for accreditation‐required supervision and to satisfy billing requirements.

The requirements also indicate that senior residents must train as the primary patient caregiver for a portion of their training, separate from their time supervising or teaching junior residents. If a program chooses to assign a senior resident to an area of the ED now staffed by an attending, or a program chooses to replace an APC with a senior resident in an area where an APC works with an attending, the APC salary costs may also be decreased. Similarly, in some institutions, an increase in the resident time in the critical care units may possibly decrease the staffing needs of these units. The ultimate financial results of these changes will be dependent upon local physician and billing practices (Figure 9).

**FIGURE 9:**
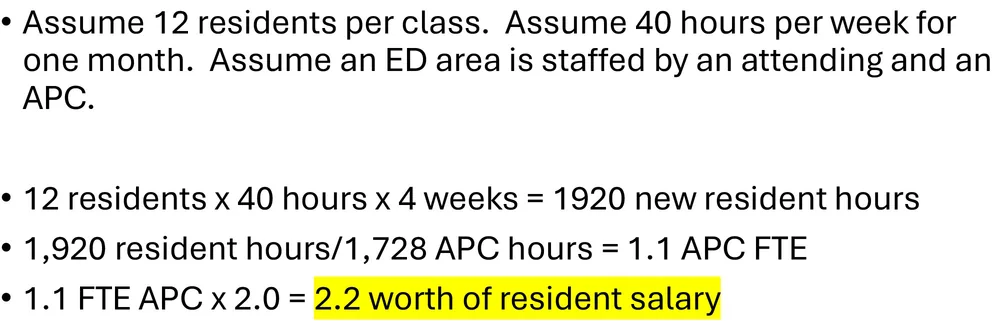
Senior resident primary patient caregiver for 4 weeks.

## 4 Understand Staffing Implications in Low Acuity and Observation Areas

It is required that residents train in lower acuity areas and an experience in observation medicine. If these areas are currently staffed by APCs, assigning senior residents to these areas instead of APCs may result in substantial cost saving of APC salary for the hospital. Conversely, junior EM residents assigned to low acuity or observation areas may be more inefficient than experienced APCs. If faculty currently staff in these areas, supervision of more than one junior EM resident may increase the billable volume seen by a single EM attending physician. Understanding local throughput and billing implications are critical to properly staffing these areas to satisfy contemplated program changes and operational imperatives (Figure 10).

**FIGURE 10:**
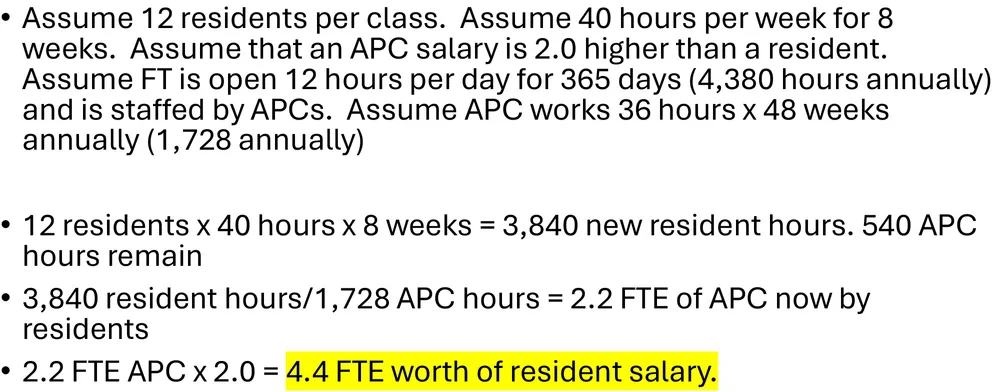
Eight weeks in low acuity area or observation.

## 5 Recover Expenses for New “Out Rotations”

Assigning residents to low‐resourced EDs that are within a hospital system might allow for savings if APCs are reduced in the low‐resourced ED or if additional resident staff facilitate ED throughput. If the low‐resourced ED is another hospital with a CMS cap, GME cap‐sharing agreements can assist a home hospital in recovering resident salary expenses. Rotations to a freestanding ED, not owned by a hospital, may be eligible for home hospital CMS reimbursement if the site is considered a “non‐provider setting.” New rotations to Critical Access Hospitals [9] or other rural‐designated hospitals, including some Veterans Administration sites, may allow the home hospital (where the residents are employed) to recover resident expenses (Figure 11).

**FIGURE 11:**
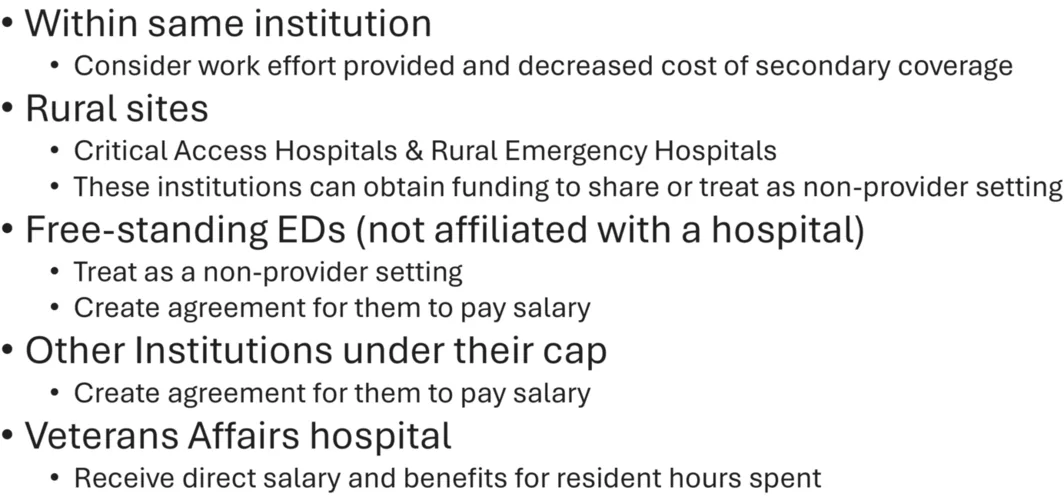
Low‐resourced ED—Possible financial arrangements.

## 6 Summary

The new EM program requirements present challenges and opportunities to programs that will lead to discussion of finances. If the pilot project leads to transitioning from a 3‐year format to a 4‐year format, this will be a major change requiring planning. Current 4‐year programs will be eligible for additional CMS funding and may find additional financial value in the implementation of new program requirements. Current 3‐year programs may choose to change their program's complement, which will be the prime determinant of program finances. A reduction in complement may provide a savings in resident salaries, but staffing required to “back fill” few residents may be more expensive than savings with a smaller complement. A program choosing to remain “complement neutral” can realize some financial benefits depending on the execution of some of the steps suggested in this paper. Programs choosing to expand the complement, particularly if the program's hospital has additional CMS cap, may find that the increased salaries related to a program expansion are easily offset by additional CMS revenue and other initiatives described here. Other changes that are required in resident experiences and responsibilities could have financial implications, and GME leaders should seek understanding of the implications at their institution.

All programmatic changes must prioritize local institutional goals and commitment to educational excellence. This concept paper focuses on a general overview of the impact on residency funding of potential approaches to the EM program requirements. That a program is eligible for funding does not guarantee funding. Each institution has its own unique CMS reimbursement and cap as well as other financial structures that impact this discussion. Each institution should confer with local leadership to prioritize patient care, educational outcomes, and understand the impact of local financial issues.

## Author Contributions

**Douglas McGee:** conceptualization, investigation, writing – original draft, writing – review and editing, formal analysis, project administration, methodology, validation, visualization, data curation, resources. **Mary Jo Wagner:** conceptualization, investigation, writing – original draft, writing – review and editing, visualization, validation, methodology, formal analysis, project administration, data curation, resources.

## Funding

The authors have nothing to report.

## Conflicts of Interest

The authors declare no conflicts of interest. Drs. D. McGee and M. J. Wagner were members of the ACGME EM 10‐year revision working group, but they have not written this as representatives of the ACGME or the volunteers on the working group. They are subject matter experts on GME funding and financing.

## Data Availability

The data that support the findings of this study are openly available in government websites for Centers for Medicare and Medicaid Services.

## References

[1] “Centers for Medicare & Medicaid Services Direct Graduate Medical Education (DGME),” accessed January 22, 2026, https://www.cms.gov/medicare/payment/prospective‐payment‐systems/acute‐inpatient‐pps/direct‐graduate‐medical‐education‐dgme.

[2] “Centers for Medicare & Medicaid Services Indirect Graduate Medical Education (IME),” accessed January 22, 2026, https://www.cms.gov/medicare/payment/prospective‐payment‐systems/acute‐inpatient‐pps/indirect‐medical‐education‐ime.

[3] Association of American Medical Colleges , “Medicare Payments for Graduate Medical Education: What Every Medical Student, Resident and Advisor Needs to Know,” 2025, accessed January 22, 2026, https://store.aamc.org/medicare‐payments‐for‐graduate‐medical‐education‐what‐every‐medical‐student‐resident‐and‐advisor‐needs‐to‐know.html.

[4] M. J. Wagner , H. A. Frazier , and J. S. Berger , “Navigating the Rapids: How Government Funds Flow to Graduate Medical Education,” Journal of Graduate Medical Education 16, no. 3 (2024): 339–340. 38882428 10.4300/JGME-D-24-00378.1 PMC11173030

[5] Code of Federal Regulations , “42 CFR 413.79(a) Direct GME Payments: Determination of the Weighted Number of FTE Residents,” accessed January 22, 2026, https://www.ecfr.gov/current/title‐42/part‐413/section‐413.79#p‐413.79(a).

[6] American Board of Emergency Medicine , “Policy on EM Residency Training Requirements,” accessed February 3, 2026, https://www.abem.org/wp‐content/uploads/2024/08/em‐residency‐training‐requirements.pdf.

[7] K. B. Smith , C. Kiefer , E. Shaver , et al., “Comparison of Direct Staffing Costs of Advanced Practice Providers and Residents in a High Acuity Area of a Community Emergency Department,” Marshall Journal of Medicine 7, no. 2 (2021): 39.

[8] M. M. Mrdutt , R. A. Weber , L. M. Burke , J. S. Thomas , H. T. Papaconstantinou , and C. T. Cable , “Financial Value Analysis of Surgical Residency Programs: An Argument Against Replacement,” Journal of Surgical Education 75, no. 6 (2018): e150–e155. 30100323 10.1016/j.jsurg.2018.07.004

[9] “Centers for Medicare & Medicaid Services MLN – Information for Critical Access Hospitals,” accessed February 3, 2026, https://www.cms.gov/files/document/mln006400‐information‐critical‐access‐hospitals.pdf.

